# Genome-Wide Identification, Characterization, and Expression Analysis of the *NAC* Gene Family in *Litchi chinensis*

**DOI:** 10.3390/genes14071416

**Published:** 2023-07-08

**Authors:** Guihua Liao, Yu Duan, Congcong Wang, Zebin Zhuang, Haishi Wang

**Affiliations:** Guangdong Academy of Forestry, Guangdong Provincial Key Laboratory of Silviculture, Protection and Utilization, Guangzhou 510520, China; a18218214987@163.com (G.L.); ysdwj01@gmail.com (Y.D.); d10801690511@163.com (Z.Z.); y8482868@gmail.com (H.W.)

**Keywords:** *Litchi chinensis*, *NAC*, evolution, collinearity, gene expression, *cis*-acting elements

## Abstract

NAC proteins play an essential role in the growth and development of litchi, especially during reproductive development. However, a comprehensive analysis of the litchi *NAC* gene family is currently absent. Based on information from the litchi genome, we found that the 112 NAC genes of litchi show an uneven distribution on the chromosomes. Phylogenetic and conserved structural domain analyses indicated that different types of variability were exhibited in the family of litchi NACs (LcNACs). Gene covariance analysis showed that the *LcNACs* showed better similarity in the same genus than with *Arabidopsis*. We further investigated the differential expression patterns of *LcNACs* in buds and rudimentary leaves of litchi. qRT-PCR results implied that they were involved in the process. Profiling of *LcNAC* promoter elements in litchi showed that they were extensively involved in light response, phytohormone regulation, abiotic stress response, and plant growth and development processes. This study provides new insights into the identification, structural characterization, tissue-specific expression analysis, and promoter response elements of *LcNACs*. It reveals the characteristics of the *LcNACs* and lays the foundation for the subsequent understanding of its biological functions and molecular regulatory mechanisms.

## 1. Introduction

NAC transcription factors are a family of plant-specific transcription factors named after the first letter of the *NAM* (no apical meristem) gene of *Petunia hybrida*, the *ATAF1/2* (arabidopsis transcription activation factor) gene, and the *CUC2* (cup-shaped cotyledon) gene of *Arabidopsis thaliana*, due to their similar conserved structural domains [1,2]. The genome-wide analysis of NAC transcription factors revealed that the number of Arabidopsis family members has grown to 117 and 41 in *Petunia* [3]. The NAC transcription factor is a protein structure that can be divided into two parts, the N-terminal part and the C-terminal part. The N-terminal part is about 150 amino acids in length and is the DNA binding domain that is responsible for binding to the cis-acting element on the promoter of the target gene. The N-terminal part is usually divided into five subdomains, A, B, C, D, and E, of which A, C, and D are highly conserved and may be associated with the presence of approved signals and conserved motifs. The B and E subdomains are variable in sequence and are associated with the functional diversity of NAC family members and are involved in many unique functions of NAC proteins [4,5,6]. The C-terminus of NAC transcription factors is the transcription regulatory region (TRR), which can activate or repress the transcription process of target genes [7]. The C-terminal sequences vary in length and are mainly composed of simple amino acids such as serine, proline, and glutamate, which are the main factors for the functional diversity of NAC family members [8]. The presence of the motif structure is also conserved for the NAC subfamily, but it differs between subfamilies, so the presence of the motif structure also enriches the function of individual NAC proteins. Normally, most NAC proteins are localized in the nucleus, but some motifs can help encode proteins localized in the membrane system of plant cells and are named NTLs (NAC with transmembrane motif 1-like, NTM1-like). When plants are stimulated by an adverse environment or other factors, NTLs will enter from the membrane system to the nucleus to regulate the expression of related genes and participate in plant growth and development [9] and biotic and abiotic stress processes [10].

The NAC transcription factor is a “molecular switch” in plant growth and development [11,12,13]. Numerous studies have shown that NAC transcription factors are involved in the regulation of plant growth and development, resistance to stress, disease resistance, and hormone signaling [10,14]. Elasad et al. discovered that the NAC transcription factor in cotton *Gossypium* positively regulates leaf senescence [15]. *NAP* is a member of the NAC family associated with senescence in *Arabidopsis*, and overexpression of the *NAP* gene resulted in early senescence, while the *NAP* mutant plants showed delayed senescence. *ORE1* is another member of the NAC family associated with senescence in Arabidopsis, and overexpression of the *ORE1* gene resulted in early leaf senescence, probably through regulation of the *SAGs* gene. Conversely, *ORE1* mutant leaves showed delayed senescence and increased resistance to oxidative stress. In addition, there are other *NACs* associated with senescence in Arabidopsis, such as *JUB1*, which can reduce the level of H_2_O_2_, inhibit the expression of senescence genes, and prolong plant lifespan. *VNI2* and *ANAC046* are involved in leaf senescence [16]. In rice leaves, *OsNAC2* can directly activate the expression of chlorophyll degradation gene *OsNYC3* and ABA degradation-related genes to promote leaf senescence. In tomatoes, *SlNAP2* can directly control the expression of senescence-related gene *SlSAG13* and chlorophyll degradation-related genes *SlSGR1* and *SlPAO*, thus regulating senescence and controlling yield. It was found that *AaNAC2*, *AaNAC3,* and *AaNAC4* in kiwifruit accumulate more terpenes by activating the expression of terpene synthase genes, resulting in softer fruit and promoting fruit ripening. *SHATTERING1-5* is a member of the soybean NAC transcription factor family, and the *SHATTERING1-5* gene promotes the synthesis of secondary cell walls in pod-joining cells, thickening the secondary walls so that the pods are less likely to dehiscence. The *NAC* acts as a regulatory factor that regulates the expression of downstream genes and is also itself regulated by other factors such as *miRNAs* [17,18,19]. *miRNA164* can suppress the excessive formation of petals and organ boundaries during *Arabidopsis* flower development by negatively regulating the expression of *CUC1* and *CUC2*. The expression of *CUC1* and *CUC2* is negatively regulated by *miRNA164* to suppress excessive petal formation and organ border expansion during *Arabidopsis* flower development [17]. With the development and widespread use of transcriptome sequencing technology, a large number of NAC transcription factors have been found to be involved in the process of plant organ abscission. For example, *NAC* genes have been found to be differentially expressed in petal delaminations at different opening stages in moonflower, involved in the process of sugarcane leaf abscission [20], regulating the formation of petiole delaminations in ripe melon fruits [21], and present in the process of ripe fruit abscission in olives and apples [22,23].

Litchi (*Litchi chinensis* Sonn.) belongs to the sapindaceae family and is nutrient-rich with high economic value [24]. Litchi is a specialty fruit of the southern part of China with a long history of cultivation. China has many good varieties of litchi, and litchi germplasm resources are abundant [25,26]. The induction process of litchi flower formation requires a specific low temperature environment [27]. If the temperature is too high, litchi flower bud development is restricted. At this time, the rudimentary leaves develop rapidly into branch tips, which seriously affects the efficiency of flower formation [28,29]. Litchi inflorescence is a panicle with a larger number of flowers, but that does not mean a greater number of fruit set. Litchi juvenile fruits are susceptible to various factors that affect the phenomenon of fruit setting [30]. With the increasing trend of global warming, throughout the reproductive growth period litchi are confronted with a number of challenges from both inside and outside [31]. The ultimate goal of scientific research is to obtain fruitful results. Therefore, litchi flower induction, fruit abscission, and eventual trans-color ripening has become a topical aspect of litchi research [32,33]. 

In response to the developmental requirements of the litchi industry, the involvement of NAC transcription factors has been found in all the growth and developmental stages involved in the current scientific research on litchi [7,34,35]. Liu et al. sequenced the expression profiles of rudimentary leaves and inflorescences at different stages of senescence and found that several differentially expressed genes were *NAC*-like transcription factors and were enriched in cellular processes, metabolic processes, and protein metabolism [36]. Jiang et al. cloned 13 *NAC* genes from litchi fruit and found that with the acceleration of fruit senescence, *LcNAC1* expression was significantly upregulated in the pericarp [35]. The expression of *LcNAC1* was significantly upregulated in the pericarp and pulp with the acceleration of fruit senescence. In the experiment of litchi fruit abscission induced by ethylene glycol treatment, Li et al. used RNA-Seq transcriptome sequencing to mine 2730 genes associated with fruit abscission, including 12 *NAC* genes [30]. Although the *NAC* gene family has been reported in a variety of other plants, a comprehensive analysis of the NAC family in litchi has not been performed. 

In this research, all *NAC* genes were screened using the genomic database based on the completion of whole genome sequencing of litchi [37]. The genetic organization of litchi NAC at the expression level and genetic level was characterized by NAC-specific structural domains. The similarities and differences between the evolutionary process of the litchi NAC family and the model plant *Arabidopsis thaliana* were analyzed by gene family evolutionary relationships [3]. With the help of gene co-linearity analysis, we analyzed the co-linearity characteristics of litchi *NAC* within the same genus and the differences between its co-linearity with *Arabidopsis NAC*. Combined with published transcriptomic data, the role of *NAC* in the processes of litchi bud shriveling and fruit abscission was explored. RT-qPCR was used to further validate the expression pattern of *NAC* during litchi bud development, providing a reference for in-depth investigation of *NAC* regulation during the litchi bud development process. The screening and bioinformatics analysis of the *LcNAC* gene family will lay the foundation for a better understanding of their gene functions. It will also provide a theoretical basis for improving the flowering and fruiting of litchi and provide the corresponding theoretical support for the regulation of litchi production.

## 2. Materials and Methods

### 2.1. Identification and Characterization of NAC Family Genes in Litchi

The *NAC* genes containing the NAM structural domain (PF02365) were gathered from the litchi genome database (http://www.sapindaceae.com/index.html accessed on 1 March 2023) using HMMER 3.0. Based on the HMMER determination, the candidate genes containing NAM structures were further manually validated using the NCBI database (https://www.ncbi.nlm.nih.gov/guide/ accessed on 1 March 2023). The NAM conserved structures of all candidate genes were further validated using the conserved structure database (CCD) [38,39], PFAM program (http://pfam.xfam.org/ accessed on 1 March 2023), and SMART program (http://smart.embl-heidelberg.de/ accessed on 1 March 2023). The PFAM database was used to identify litchi NAC family genes using the PF02365 identifier with an e value < 1e^−4^ [40,41]. The above results were combined to identify the obtained *LcNACs* for subsequent analysis. 

We used the tools on the ExPasy website (https://www.expasy.org/ accessed on 1 March 2023) to obtain the molecular weight (MW) and isoelectric point (pI) of the *LcNACs* [42,43]. Meanwhile, the chromosomal localization and predicted coding amino acid (aa) length of the *LcNACs* were obtained using the litchi genome database. The *NAC* sequences of *A. thaliana* were downloaded from the TAIR database (https://www.arabidopsis.org/ accessed on 1 March 2023). The most similar homologous NACs to *Arabidopsis* were obtained by Blastp comparison (https://www.arabidopsis.org/Blast/index.jsp accessed on 1 March 2023). Prediction of subcellular localization of litchi NAC proteins were predicted using SPORT (https://psort.hgc.jp/ accessed on 1 March 2023).

### 2.2. Phylogenetic Analysis and Classification of LcNACs

The NAC protein sequences of *Arabidopsis* used for evolutionary tree construction were downloaded from the plant transcription factor database (http://planttfdb.gao-lab.org/ accessed on 1 March 2023). In this study, the maximum resolution method (MP) in MEGA 7.0 was used for the phylogenetic tree construction of the *LcNAC* gene family [44]. First, the *NAC* gene-encoding proteins of litchi (112) and *Arabidopsis* (138) were aligned using ClustalW (version 2.0) software. Then, multiple sequence alignments were recovered in PHYLIP format, and a phylogenetic tree was selected to be constructed using the maximum likelihood. The bootstrap replications of the evolutionary tree were set to 1000 to estimate the accuracy. EvolView (https://itol.embl.de/) was then used to display the uneradicated phylogenetic tree [45].

### 2.3. Gene Structure, Conserved Motif Analysis, and Chromosomal Location of LcNACs

The boundary display constructs were constructed using the gene structure display server (GSDS, S, http://gsds.gao-lab.org/ accessed on 1 March 2023) based on the available CDS sequences of the *LcNACs* and the litchi genome sequence [46]. Conserved motif analyses of *NAC* genes were performed using the multiple expectation maximization for motif elicitation program (MEME, http://meme-suite.org/tools/meme accessed on 1 March 2023). We retrieved the chromosomal localization of each NAC gene in the litchi genome general feature format (gff) to obtain the chromosome number, start position, and end position. The chromosome positions of *LcNACs* in the common feature format file were annotated. This chromosome information file was then submitted using MapChart software (version 2.32) to display the distribution of each gene [47].

### 2.4. Chromosomal Mapping, Gene Duplication, and Synteny Analysis

The data used for the co-linearity analysis between species were obtained from the sapindaceae genomic database (http://www.sapindaceae.com/index.html accessed on 1 March 2023, including litchi chinensis, Dimocarpus longan, Xanthoceras sorbifolium, Nephelium lappaceum, Acer yangbiense) and TAIR databases (including Arabidopsis thaliana). After obtaining the genome files and gff files of several species, the multiple covariance scanning toolkit (MCScanX) was used to obtain the chromosome distribution of the NAC family using default parameters [48,49]. The dual synteny plotter of TBtools (https://github.com/CJ-Chen/TBtools accessed on 1 March 2023) was used to analyze the homology of the *NAC* genes between litchi and the other plants [50]. 

### 2.5. Prediction of Cis-Acting Elements within the Promoter of NAC Genes in Litchi

Based on the litchi genome sequence information, the 2000 bp sequence upstream of the *LcNACs* was extracted and used as a promoter sequence for cis-acting element analysis. The PlantCare tool (http://bioinformatics.psb.ugent.be/webtools/plantcare/html/ accessed on 1 March 2023) [51] was used to determine the cis-acting elements of *LcNAC* promoter sequences.

### 2.6. Expression Profiling of Litchi NAC Genes

To compare the expression of *LcNACs* in various tissue sites, we explored the raw data of *NAC* gene expression in various tissues of litchi from the sapinaceae genomic database (http://www.sapindaceae.com/Gene-Expression-V2/GeneExpression.html accessed on 1 March 2023) using an interactive cartoon heat map and presented it in the form of a heat map [52,53]. To investigate the expression pattern of *NAC* genes involved in flower bud development and rudimentary leaf senescence in litchi, we screened differentially expressed *NAC* genes from transcriptomic data from previous studies of competitive development of litchi flower buds and rudimentary leaves. The transcriptome data from different tissues were obtained through the NCBI short read archive database (https://www.ncbi.nlm.nih.gov/sra/ accessed on 1 March 2023) (accession number: PRJNA430479). The *NAC* genes were screened based on the criterion of more than 2-fold change in the mean FPKM value of *NAC* gene expression and presented in the form of heat map. Heat map analysis was performed using OmicShare tools, a free online platform for data analysis (www.omicshare.com/tools accessed on 1 March 2023).

### 2.7. Plant Materials and Growing Conditions of Litchi

Seven-year-old ‘Nuomici’ litchi trees were planted in 40 L pots with a substrate containing grass charcoal and sandy loam soil (*v*/*v*, 1:3). Potted plants were placed under natural winter conditions for floral induction. When panicle induction was successful at first appearance, 3 litchi plants were screened and transferred to a temperature-controlled greenhouse set to a 12 h photoperiod and 18 °C to promote panicle development and promote senescence of rudimentary leaves. An additional 3 litchi plants were designed to be transferred to a growth chamber with a 12 h photoperiod and a temperature of 26 °C to encourage rudimentary leaf growth and inflorescence shrinkage. For gene expression assays, developing flower buds and senescence rudimentary leaves at low temperature in stage 5 and shriveled flower buds and normal developing rudimentary leaves at high temperature in stage 5 were collected as described by Yang et al. [29]. They were rapidly frozen in liquid nitrogen and stored at −80 °C for subsequent total RNA extraction and gene expression assays.

### 2.8. Quantitative Real-Time PCR Analyses of NAC Expression Patterns

RNA-seq data of differentially expressed *NAC* genes during competition between litchi flower buds and rudimentary leaf development were validated by quantitative real-time PCR (qRT-PCR). The primer Express 5.0 [54] was used to design qRT-PCR gene-specific primers. Total RNA was extracted using the total plant RNA kit (Huayueyang, Beijing, China). The cDNA was transcribed from mRNA using the PrimeScript cDNA synthesis kit (TaKaRa, Dalian, China). The reverse-transcribed cDNA was used as a template for the qRT-PCR. The qRT-PCR was performed using the CFX96TM real-time system (Bio-Rad, Beijing, China) combined with sequence-specific primers (Supplementary Table S1). Three biological replicates were designed for each gene, and the cycling conditions set were: denaturation at 95 °C for 5 s, annealing at 55 °C for 15 s, followed by extension at 65 °C for 5 s. Data analysis was performed using the 2^−ΔΔCt^ method to calculate the expression levels of transcripts [55,56].

## 3. Results

### 3.1. The Litchi Genome Contains 112 NAC Genes and Is Unevenly Distributed on 15 Chromosomes

The NAC family members in the litchi genome were identified using a hidden Markov model (HMM) with a genome-wide search for genes containing the NAM structural domain. We obtained a total of 112 *NAC* genes based on the presence or absence of NAM structural domains (Supplementary Figure S1) and renamed them *LcNAC1* to *LcNAC112* according to their position on the chromosomes. For easy retrieval, each renamed *NAC* gene symbol corresponds to the published sequence ID of each gene in the litchi genome. In this study, the identified litchi *NAC* genes were analyzed for basic information, including the detailed position of each *NAC* gene on the chromosome, the length of the predicted *NAC* gene coding sequences (CDS) that may encode amino acids and their relative molecular weight (MW), and the isoelectric point (pI). The predicted protein sequence lengths of the NAC genes in litchi ranged from 63 (LcNAC29) to 719 (LcNAC37) amino acids. The MW range was between 7145.02 and 81384.92, and the pI values ranged from 9.63 to 4.23 with a mean value of 6.78, indicating that most of the proteins of the *NAC* gene in litchi were weakly acidic (Supplementary Table S2).

**Figure 1:**
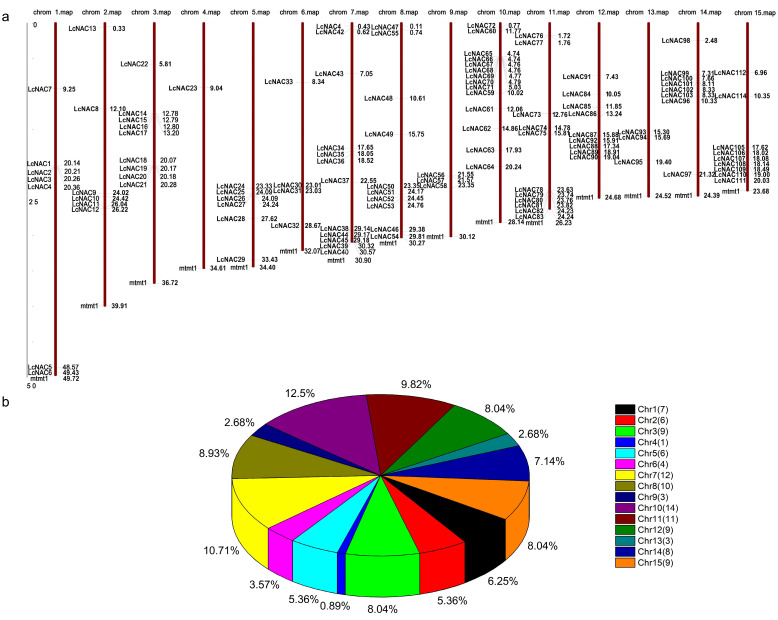
Chromosome location of *LcNACs* on each chromosome of litchi chinensis. (**a**) Schematic representation of the distribution of *NAC* genes on each chromosome (red bars). The name of each *NAC* gene contained on the chromosome is shown on the left side of the chromosome. The approximate location of each *NAC* gene is shown to the right of each chromosome. (**b**) Distribution statistics of *NAC* genes per chromosome. The number of *NAC* genes on each chromosome is shown in brackets in the upper right icon. The percentage of each chromosome containing the number of *NAC* genes is shown next to the pie chart color block.

Analysis of the location of the *NAC* genes on the chromosomes of litchi revealed that the *NAC* genes were distributed on all 15 chromosomes (Figure 1a). Gene distribution was uneven between chromosomes. Among them, chromosome 10 contained 16 *NAC* genes, which was the chromosome with the largest number of *NAC* genes, while chromosome 4 contained only 2 *NAC* genes, which was the least number (Figure 1b).

### 3.2. Gene Phylogenetic Analysis, Gene Structure, and Protein Motif Composition Analysis

**Figure 2:**
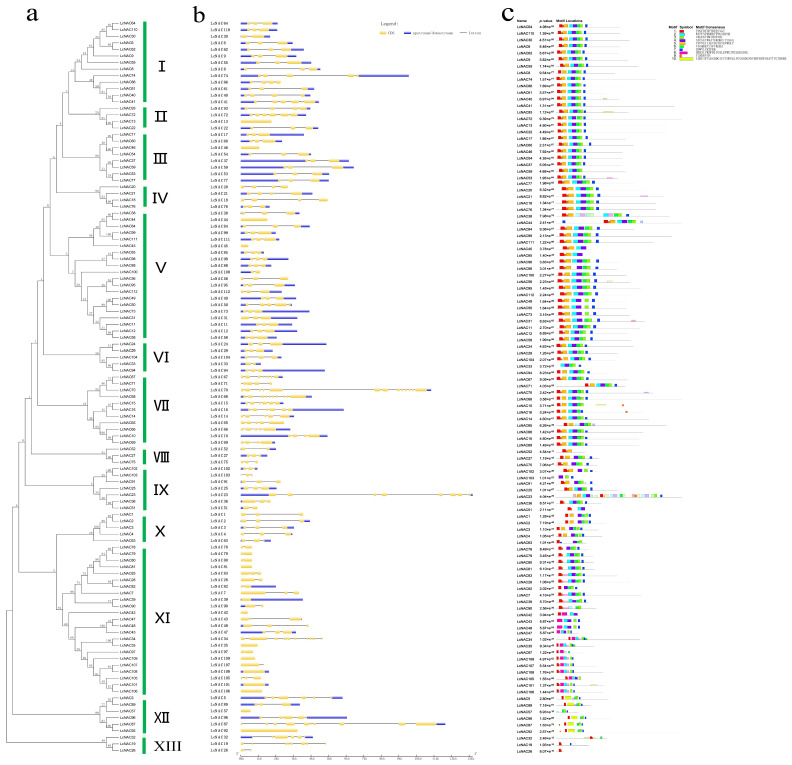
Unrooted phylogenetic tree, genomic sequence structure, and conserved motif analysis of protein sequences of the *LcNACs*. (**a**) The neighbor-joining tree on the left is constructed from 112 *LcNAC* proteins. The subgroups (Groups Ⅰ-XIII) are listed on the right side of the phylogenetic tree. (**b**) Yellow color indicates the *LcNAC* sequence coding region (CDS), purple color indicates untranslated 5’ and 3’ regions, and black solid line indicates introns. (**c**) The 10 conserved motifs are indicated by different colored boxes.

To further explore the conserved relationships of *NAC* family genes in litchi, we performed multiple sequence alignment of 112 LcNAC protein sequences using Mega 6.0 software, and the results of the alignment were used to construct a phylogenetic tree according to the neighbor-joining (N-J) method. The phylogenetic tree results showed that the 112 NAC proteins were classified into 13 subgroups (Figure 2a: Groups I-XIII), and among these 13 subgroups, group “XI” contained the largest number of 23 genes. The smallest subgroups were groups “VIII” and “XIII”, each of which contained only three sequences. In terms of evolutionary relationships, there is a relatively significant difference between group XIII and XII. Group XIII and XII were significantly divided into two major branches throughout the evolutionary tree, implying that there may be relatively large differences in gene function.

To further investigate the structural composition and functions of the 112 NAC genes, we obtained the conserved coding region sequences and exon/intron positions of the NAC genes using the litchi genome sequence information. The sequences of the coding region of the NAC gene were compared with the corresponding genomic sequences using the GSDS program. The results of GSGD analysis showed that the number of exons of 112 NAC sequences ranged from 1 to 14 (Figure 2b). To gain more insight into the evolution of the litchi NAC family, we analyzed all identified *LcNACs* for structural characterization. As shown in Figure 2b, among the *LcNACs*, 10 genes (approximately 8.93%) were intronless and 12 (approximately 10.71%) had one intron. Among them *LcNAC34* with 14 exons had the highest number. Most of the sequences have three to four exons and show similar numbers within the same subgroup. The whole number of exons was in relatively good concordance with the subtrees classified by the phylogenetic tree (Figure 2a), which also indicated the similarity of the evolutionary relationships of the genes.

For the coding protein sequences, we analyzed the protein sequences encoded by the *LcNACs* using MEME software (4.11.3). We set the initial retrieval of 10 motifs for all sequences, and the final obtained motifs ranged from 8–50 amino acids in length. In addition, the information related to the 10 motifs is shown in the Supplementary Figure S2. The results showed that LcNAC26 contained only two patterns and was the gene containing the least number of pattern sequences. Although the search was set to retrieve 10 motifs, the motif containing the most motifs had only 8 (Figure 2c). However, from the overall results, the distribution of motifs showed some correlation between sequences, especially with evolutionary tree grouping showing high consistency, which supports the view that gene sequences are conserved in evolutionary relationships. More experiments are needed to verify whether the functions presented by the related genes and proteins are similar.

### 3.3. Phylogenetic Analysis of the LcNACs and A. thaliana NAC Genes

**Figure 3:**
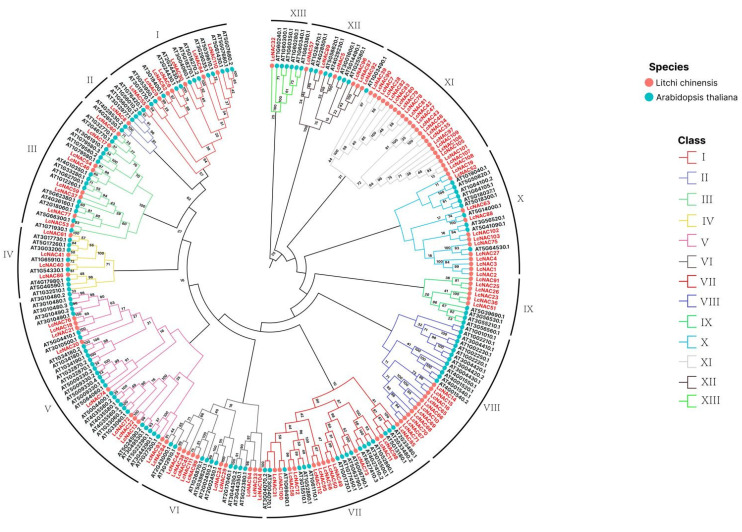
Phylogenetic analysis of NAC in *A. thaliana* and litchi. The phylogeny of the *NAC* genes of litchi and Arabidopsis is based on the amino acid sequence of NAC. The black font at the periphery of the circular evolutionary tree indicates the Arabidopsis NAC gene ID, and the red font indicates the litchi NAC gene number. Evolutionary tree subfamily branches are indicated by solid lines of different colors (I–XII). Confidence values are marked on the evolutionary tree branches.

To explore the evolutionary relationship between litchi and *Arabidopsis* NAC-encoded proteins, we obtained 138 NAC gene-encoded protein sequences of *Arabidopsis* and 112 NAC gene-encoded protein sequences of litchi for the construction of the evolutionary tree. Phylogenetic tree analysis showed that the NACs of litchi and Arabidopsis were divided into 12 sub-groups (I–XII), and the NACs of litchi showed uneven distribution in each sub-group (Figure 3). It is more apparent that within subfamily III, only one *Arabidopsis* NAC clustered with the 23 NACs of litchi. In addition, we found that in sub-groups V and VI, although within the same sub-group, litchi NAC and Arabidopsis NAC were again divided into different separate groups, indicating a possible differentiation in evolutionary relationships. 

### 3.4. Collinearity Analysis of the LcNACs

**Figure 4:**
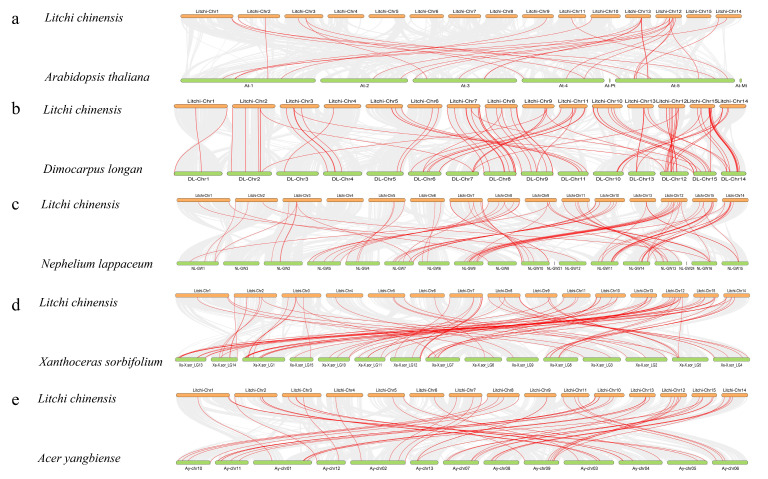
Analysis of co-linearity of *NAC* genes between litchi and five representative plant species. The gray lines in the background indicate adjacent blocks in the genomes of litchi and other plants while the red lines highlight co-linear *NAC* gene pairs. (**a**–**e**) show the covariance of *NAC* genes between litchi and five representative plants, respectively.

To further analyze the evolutionary relationships of the NAC gene family of litchi, we screened four plants in the same family as litchi and constructed a comparative synteny map of their *NAC* gene family. These four plants were *D. longan*, *N. lappaceum*, *X. sorbifolium,* and *A. yangbiense*. In addition, the *NAC* gene family of *A. thaliana* was also selected as a model plant for the construction of the comparative synteny map (Figure 4a). There were 27 *LcNACs* with co-linearity with *Arabidopsis*, *D. longan* (91) (Figure 4b), *N. lappaceum* (79) (Figure 4c), *X. sorbifolium* (81) (Figure 4d), and *A. yangbiense* (53) (Figure 4e). Compared with the *LcNACs*, only 27 *NAC* homologs were found in the *Arabidopsis* genome while 91 *NAC* homologs were found in the longan genome. This suggests that that longan and litchi are genetically closely related.

### 3.5. Analysis of Cis-Regulatory Elements of the LcNACs

The promoter regions of 112 *LcNACs* were analyzed by obtaining 2000 bp sequences upstream of the start codon and a search for predictive biotic and abiotic stress response elements. The prediction results showed that in addition to containing basic cis-elements, such as CAAT-box and TATA-box, hormone-responsive, light-responsive, plant growth and development-related, and stress-related elements were also predicted. The analysis showed that 109 genes were predicted to potentially contain hormone-responsive cis-elements. There was a total of 110 genes with light-responsive cis-elements, 103 genes with plant growth and development-related cis-elements, and 108 genes with stress-related cis-elements (Supplementary Table S3).

**Figure 5:**
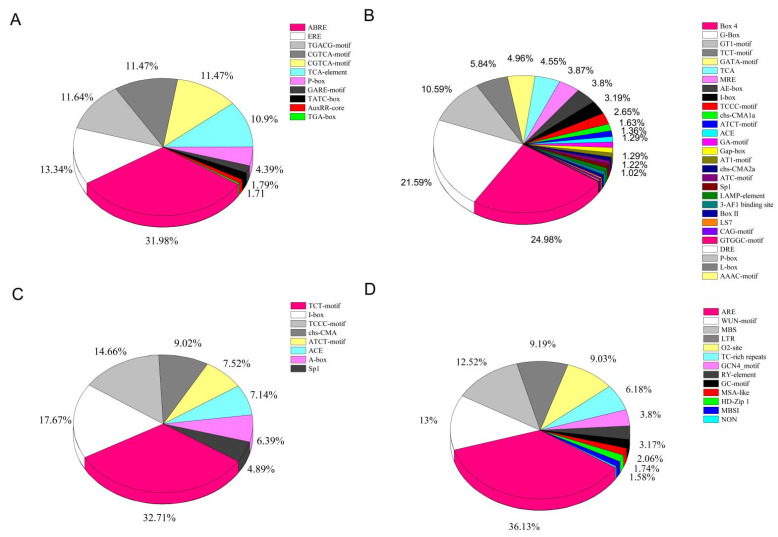
Analysis of the cis-acting elements found in the *LcNACs*. (**A**) Hormone responsive, (**B**) light responsive, (**C**) plant growth and developmental, and (**D**) stress responsive. Different colors indicate different cis-acting elements and the proportion of cis-acting elements present in the promoter of the *LcNACs*.

The predicted cis-elements in the *LcNACs* were hormone responsive, including ABRE, ERE, CGTCA-motif, TGACG-motif, TCA-element, P-box, TGA-element, GARE-motif, AuxRR-core, TATC-box, and TGA-box. Among the 11 classes of predicted cis-elements responding to hormones, ABRE, ERE, CGTCA-motif, TGACG-motif, and TCA-element were the most numerous, with the largest number of 393 ABRE elements (Supplementary Table S4) (Figure 5A). The promoter of the litchi NAC gene may respond to a total of six types of hormone signals, namely auxin, gibberellin, salicylic acid, abscisic acid, MeJA, and gibberellin. Among them, the predicted elements responding to abscisic acid and MeJA are the most frequently found. There were 40 gene promoter sequences that may be responsive to auxin, gibberellin (62), salicylic acid (49), abscisic acid (87), MeJA (73) (Supplementary Table S5). Among them, the promoters predicted to be potentially responsive to abscisic acid and MeJA had the highest number, a result consistent with the statistical type of cis-elements responding to each type of hormone (Supplementary Table S6). Light-responsive cis-elements included Box 4, G-box, GT1-element, TCT-element, GATA-motif, MRE, AE-box, I-box, TCCC-motif, chs-CMA, ATCT-motif, ACE, GA-motif, and so on. The Box 4 and G-Box types of cis-elements are the most abundant among the promoters that may respond to light reactions (Figure 5B). The cis-elements associated with development in the analysis were TCT-motif, I-box, chs-CMA, TCCC-motif, ATCT-motif, ACE, A-box, and Sp1. The most numerous of them was TCT-motif (Figure 5C). The stress-related cis-elements included ARE, MBS, WUN-motif, LTR, O2-site, TC-rich repeats, GCN4-motif, RY-elements, GC-motif, MSA-like, HD-Zip 1, MBSI, and NON (Figure 5D). The most abundant element was ARE (228), followed by WUN-motif (82) and MBS (79) (Supplementary Table S4). MYB binding site element MBS is involved with drought induction. WUN-motif is closely related to stress responses, such as anaerobic and drought induction, low temperature stress, defense, and additionally to wound-responsive elements and hypoxia specific induction. The presence of such a cis-acting element in the litchi NAC promoters implies the possible existence of a corresponding regulatory function, but this requires verification by subsequent experiments.

### 3.6. Spatiotemporal Expression Analysis of NAC Genes in Different Tissues of Litchi

**Figure 6:**
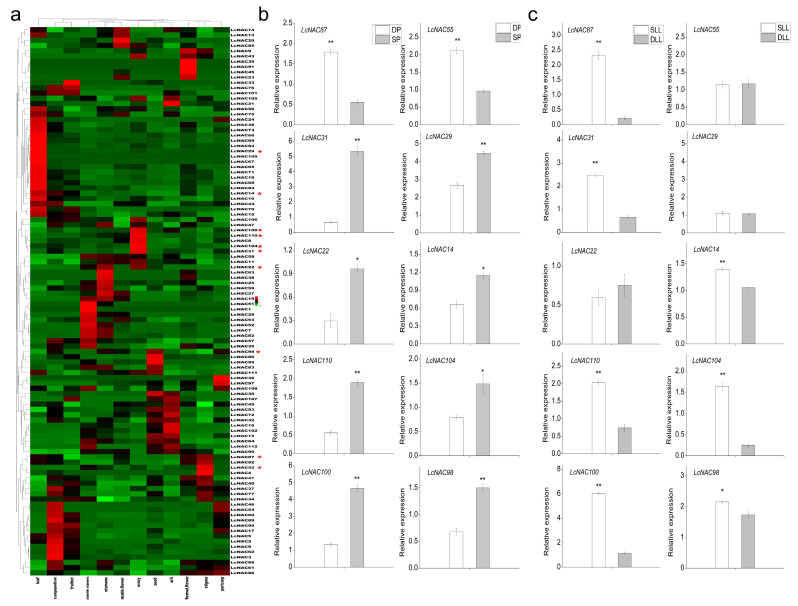
Analysis of the expression pattern of *LcNACs*. (**a**) Tissue-specific gene expression patterns of 112 *LcNACs* were analyzed using heat maps. There were 112 *LcNACs* in the carpopodium, aril, seed, pericarp, ovary, sterile stamens, leaf, stamens, stigma, female flower, male flower, and fruitlet. Data were normalized to the Z-score. The color scale illustrates the log2, and red and green show high or low transcript abundance, respectively. * represents the genes selected for the subsequent qRT-PCR. (**b**) Expression of 10 candidate *LcNACs* within developing flower buds under low temperature conditions and shrinking flower buds under high temperature conditions. (**c**) Expression of 10 candidate *LcNACs* during the low temperature-induced rudimentary leaf senescence stage and the high temperature-promoted rudimentary leaf development stage. X-axis and Y-axis represent the sampling stage and relative gene expression levels, respectively. Error bars represent the standard deviation (SD) of three independent biological replicates. Asterisk (*) indicates significant difference according to Student’s *t*-test. * indicates significant differences (*p* ≤ 0.05) and ** indicates highly significant differences (*p* ≤ 0.01).

First, a heat map of *NAC* gene expression in different tissue sites of litchi was constructed to explore the potential function of the *LcNACs*. We collected the expression of *LcNACs* inside the leaf, carpopodium, pericarp, fruitlet seed, flower, and ovary of litchi. Heat maps were used to analyze the expression of *LcNACs* (Figure 6a). The results showed the differential expression of *LcNACs* in different tissues. To detect the expression pattern of *LcNACs* in developing and shrinking flower buds in response to temperature changes, we screened differentially expressed *NAC* genes using transcriptome data and compared their expression patterns by heat map construction. The expression patterns of differentially expressed *NAC* genes were analyzed using qRT-PCR using developing panicles (DPs) under low temperature (LT) and shrinking panicles (SPs) under high temperature (HT) conditions (Figure 6b,c). The results showed that *LcNAC87* (*LITCHI020609*) and *LcNAC55* (*LITCHI009913*) showed a downregulated expression trend in SPs, while the other *LcNACs* showed an upregulated expression trend, indicating that most differentially expressed *NAC* genes were involved in the SP stage and might play an important role in promoting the DP stage (Figure 6b).

The differentially expressed *LcNACs* in flower buds were analyzed by qRT-PCR for expression patterns using developing leaves at a later stage under high temperature (DLL) and senescing leaves at a later stage under low temperature (SLL), which correspond to the developmental state of flower buds. The results showed that *LcNAC55* (*LITCHI009913*) and *LcNAC29* (*LITCHI002109*) did not show significant expression differences in the SLL and DLL stages. *LcNAC87* (*LITCHI020609*) showed a high expression pattern in both SLL and DP, suggesting that the high expression of *LcNAC87* may promote the development of litchi flower buds and promote the senescence of litchi rudimentary leaves. In addition, *LcNAC31* (*LITCHI003437*), *LcNAC14* (*LITCHI026762*), *LcNAC110* (*LITCHI019504*), *LcNAC104* (*LITCHI018901*), *LcNAC100* (*LITCHI005204*), and *LcNAC98* (*LITCHI004596*) showed high expression patterns in both SLL and SP, suggesting that all these *LcNACs* may be involved in the senescence process of litchi tissue organs (Figure 6c). 

### 3.7. Analysis of the Role of the NAC Genes in Floral Bud Differentiation in Litchi

**Figure 7:**
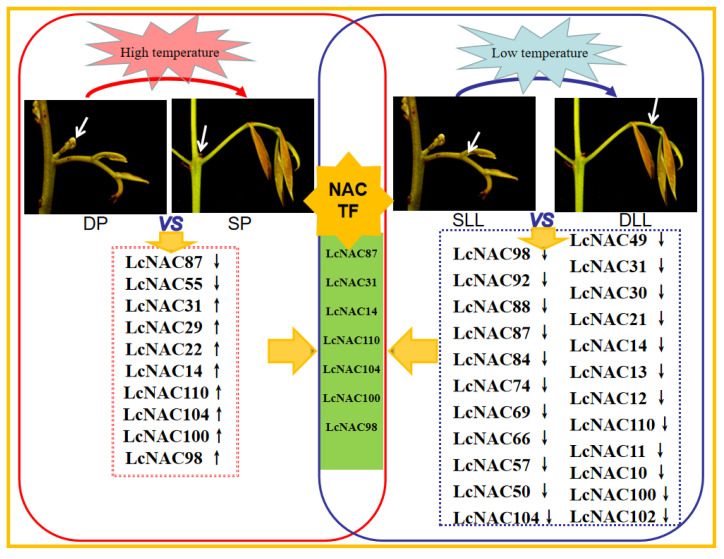
A model for summarizing the role of NACs based on the gene expression profile of litchi. DP stands for developing panicles under low temperature; SP stands for shrinking panicles under high temperature; DLL stands for developing leaves at a later stage under high temperature; SLL stands for senescing leaves at a later stage under low temperature.

Temperature is an important factor affecting competition between flower buds and rudimentary leaves in litchi. In the study of temperature-influenced flower buds and rudimentary leaf competition development, NAC transcription factors were found to be extensively involved and one of the important factors determining flower bud development (Figure 6). When the litchi flower buds finish differentiating, the litchi flower buds are mixed buds with inflorescence and rudimentary leaves coexisting, which receive the influence of external temperature and develop in different directions (Figure 7). In the transcriptional data of this process, an important regulatory role played by NAC transcription factors was found. In addition, a significant amount of NAC was involved in the temperature-influenced premature leaf senescence of litchi. The *NAC* is also involved in the process of flower bud development. Among the DP and SP differentially expressed *LcNACs*, 70% of them (*LcNAC87*, *LcNAC31*, *LcNAC14*, *LcNAC110*, *LcNAC104*, *LcNAC100*, *LcNAC98*) also showed differential expression in the development of rudimentary leaves in SLL and DLL. Further analysis of the response patterns of NAC transcription factors in temperature-influenced litchi flower bud development is important for studying the regulation of litchi flower formation. 

## 4. Discussion

NAC is a family of transcription factors widely present in plants and is unique to terrestrial plants with numerous family members [3,4,57]. With the rapid development of modern molecular biology techniques, the specific functions of NAC transcription factors have been gradually discovered. Originally, three genes were identified, *P. hybrida NAM* (no apical meristem), *A. thaliana ATAF1/2* (Arabidopsis transcription activation factor), and *CUC2* (cup-shaped cotyledon), which were named NAC transcription factors after their initials because they all had similar conserved structures. The current genome-wide analysis of NAC transcription factors identified 113 NAC members in *Arabidopsis*, 41 in *Petunia*, and 227 in *Nicotiana benthamiana* [11,58]. NAC transcription factors were also identified in many fruit trees, with apple (*Malus domestica*) having the highest number of 253 transcription factor members [4], followed by white pear (*Pyrus bretschneideri*) and sand pear (*Pyrus pyrifolia*), both with 185 members [59], while the herbaceous fruit trees *Ananas comosus* and *Carica papaya*, as well as *Vitis vinifera* [60], had fewer NAC family members with 73, 82, and 70, respectively. NAC transcription factors play an important role in plant growth and development. Most plant *NAC* family genes were obtained by screening, which has laid an important foundation for NAC gene function studies [61,62,63,64]. Studies have demonstrated that NAC transcription factors play an essential role in plant growth and development [65], stress response [66], fruit development [9], plant senescence [67] and fruit ripening [9,61].

We screened the litchi *NAC* gene family based on the data of the litchi genome sequencing analysis and initially identified the members of the litchi NAC family. Based on this foundation, we analyzed the chromosome distribution, gene structure, evolutionary tree, co-linearity of NAC genes in plants within the same family, and the expression of some NAC genes in litchi. NAC transcription factors perform regulatory functions by relying on a highly variable C-terminal transcriptional regulatory structural domain. Similar to the model plant Arabidopsis, the protein sequence of the litchi NAC transcription factor family consists of an average of about 350 amino acids with typical and non-typical NAC structural domains (Figure 2). In the present study, a phylogenetic tree of the NAC proteins of litchi and Arabidopsis was constructed based on the similarity of amino acid sequences. A distinct grouping was still formed between litchi NAC and Arabidopsis in some subgroup evolutionary relationships. It indicated that these subgroups exhibit significant differences between herbaceous and fruit tree NAC transcription factors in terms of internal evolutionary relationships. Certainly, the woody plant litchi NAC transcription factors showed evolutionary diversity, providing a genetic foundation for the diversification of their function. The present study in litchi indicated that NAC is involved in multiple growth and development processes, such as litchi bud differentiation, rudimentary leaf senescence, abscission of young fruits, fruit softening and ripening, and fruit pericarp coloration.

In this project, chromosomal localization of *LcNACs* showed that the *LcNACs* were not distributed evenly on all chromosomes. We screened the model plant *A. thaliana* and four other plants in the same genus as litchi, including *D. longan*, *N. lappaceum*, *X. sorbifolium*, and *A. yangbiense*, and used co-linearity analysis to analyze the collinearity of NAC genes between species (Figure 4). The results showed that the distribution of NAC genes on 15 chromosomes of litchi had the highest number of co-linear *NAC* genes with both *D. longan* and *X. sorbifolium*, followed by *N. lappaceum* and *A. yangbiense*. In contrast, the analysis of covariance with the model plant *Arabidopsis* showed the least number of covariate *NAC* genes, which also indicates the evolutionary distancing of the NAC family between herbaceous and woody plants.

We analyzed the promoter sequence 2000 bp upstream of the 112 *NAC* gene start codon (ATG) in litchi. The differences in the types and numbers of cis-acting elements among the *LcNACs* suggested that the genes may have generated new functions while preserving their original functions during the evolutionary process, resulting in more extensive biological activity of the *LcNAC* proteins in the regulation of various physiological processes. Four types of cis-acting elements associated with light response, plant hormone regulation, abiotic stress response, and plant growth and development were predicted. Interestingly, 110 LcNACs contained light-response response elements, and only *LcNAC64* and *LcNAC72* were absent. We analyzed the promoter sequence 2000 bp upstream of the start codon (ATG) of the 112 *LcNACs*. Four types of cis-acting elements associated with light response, plant hormone regulation, abiotic stress response, and plant growth and development were predicted. The total number of additional genes involved in plant hormone regulation, abiotic stress response, and plant growth and development were all above 100. All these results indicate that the *LcNAC* family is extensively involved in the response to stress and in the regulation of growth and development of litchi. In the analysis of response to plant hormone action elements, the number of genes responding to abscisic acid action elements was the highest, which also suggested that *LcNACs* play an important function in regulating the process of rudimentary leaf senescence or flower bud shrinkage and abscission.

Previous studies have shown that NAC transcription factors are involved in the processes of litchi rudimentary leaf senescence [68,69], fruit abscission [70], and flower bud shrinkage and abortion. Based on a bioinformatics analysis, we conducted a comprehensive analysis of the *LcNAC* family genes. This is important for us to explore the screening of important *LcNACs* involved in different growth and developmental regulatory processes of litchi [36]. In this research, the *LcNACs* involved in the process of litchi bud shrinkage were identified by transcriptome differential expression analysis combined with qRT-PCR experiments to validate the analysis. The experimental validation results were in general agreement with the bioinformatics analysis results, which also emphasized the important auxiliary significance of bioinformatics analysis for experiments. The reliability of transcriptome data analysis will help us to screen the target genes more precisely. The bioinformatic analysis provides an important reference for experimental validation in exploring the different regulatory mechanisms of litchi development.

## 5. Conclusions

In this study, we identified 112 *LcNAC* family members based on the conserved structural domains specific to the *NAC* gene family using published litchi genome data. To further understand the structure and function of *LcNAC* family genes, we performed a detailed analysis of each gene structure, including gene constructs, protein-conserved motifs, and cis-acting elements of promoters. The evolution and expansiveness of *LcNACs* were investigated by performing a *NAC* gene co-linearity analysis with the model plant *A. thaliana* and plants within the same genus. In addition, we combined transcriptome data to screen 10 *LcNACs* for relative expression analysis in temperature-influenced flower bud development and shrinkage samples. This provides a reference for further validation of the accuracy of transcriptome data and further investigation of the regulatory functions of *LcNACs* involved in the developmental process of litchi flower buds.

## Data Availability

Not applicable.

## Supplementary Materials

The following supporting information can be downloaded at: https://www.mdpi.com/article/10.3390/genes14071416/s1, Supplementary Figure S1: Prediction of the NAM structural domain contained in each of the 112 litchi NAC genes. Supplementary Figure S2: Prediction of conserved motifs in litchi NAC protein sequence. Supplementary Table S1: qRT-PCR gene-specific primers; Supplementary Table S2: The details of *NAC* genes in litchi genome; Supplementary Table S3: Statistics of the four major cis-acting elements of the litchi NAC gene promoter; Supplementary Table S4: Statistics on the grouping of promoter elements of the litchi NAC gene; Supplementary Table S5: NAC genes of litchi containing hormone-responsive elements; Supplementary Table S6: Statistics on the number of hormone-like promoter response elements.
