# Supplementary material for: Genome-Wide Identification, Characterization, and Expression Analysis of the NAC Gene Family in Litchi chinensis
Source: Genes (Basel). 2023 Jul 8;14(7):1416. doi: 10.3390/genes14071416 (PMC10379382; doi:10.3390/genes14071416)
Supplement: Supplementary file 1 [file genes-14-01416-s001.zip › Supplementary Figure -S1-S2.pdf]

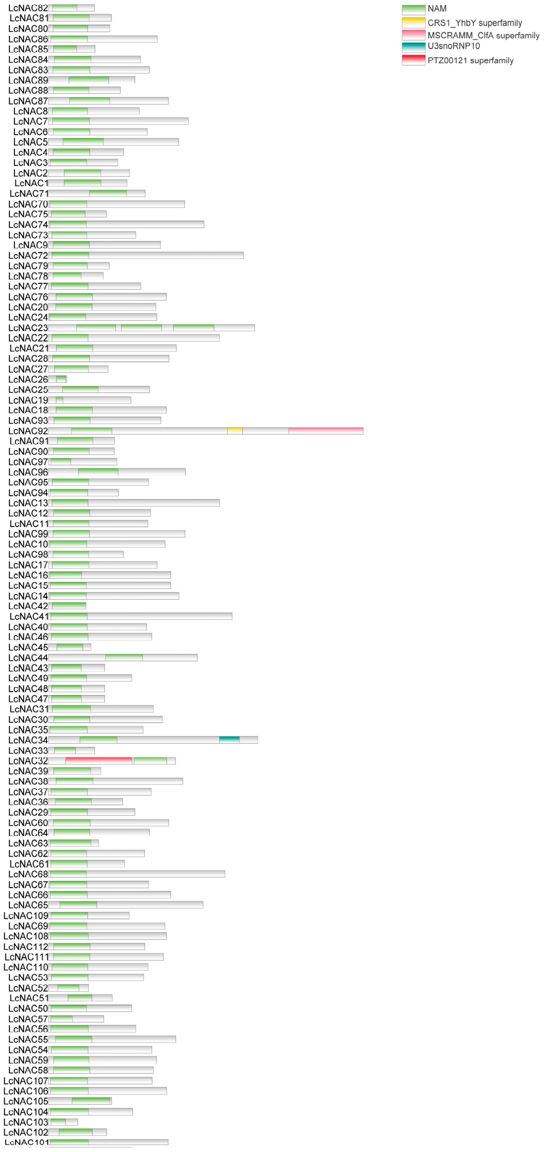

Figure S1. Prediction of the NAM structural domain contained in each of the 112 litchi NAC genes.

Discovered Motifs

	Logo	E-value	Sites	Width
1.	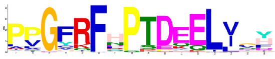	1.5e-959	106	16
2.	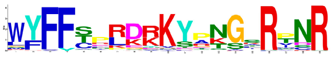	7.4e-898	86	19
3.	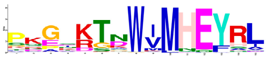	8.5e-851	102	15
4.	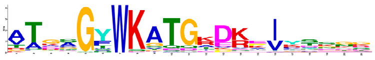	3.4e-733	92	21
5.	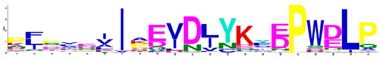	2.5e-655	75	21
6.	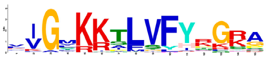	1.1e-566	91	15
7.	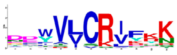	9.8e-400	99	11
8.	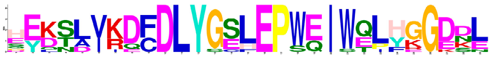	1.0e-182	13	19
9.	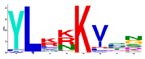	3.1e-156	84	8
10.	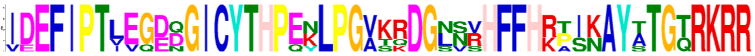	7.8e-107	5	50

Figure S2. Prediction of conserved motifs in litchi NAC protein sequence.
